# How common is clinically inactive disease in a prospective cohort of patients with juvenile idiopathic arthritis? The importance of definition

**DOI:** 10.1136/annrheumdis-2016-210511

**Published:** 2017-04-07

**Authors:** Stephanie J W Shoop-Worrall, Suzanne M M Verstappen, Eileen Baildam, Alice Chieng, Joyce Davidson, Helen Foster, Yiannis Ioannou, Flora McErlane, Lucy R Wedderburn, Wendy Thomson, Kimme L Hyrich

**Affiliations:** 1 Arthritis Research UK Centre for Epidemiology, Division of Musculoskeletal and Dermatological Sciences, Faculty of Biology, Medicine and Health, The University of Manchester, Manchester, UK; 2 NIHR Manchester Musculoskeletal Biomedical Research Unit, Central Manchester University Hospitals NHS Foundation Trust and University of Manchester Partnership, Manchester, UK; 3 Paediatric Rheumatology, Alder Hey Children's NHS Foundation Trust, Liverpool, UK; 4 Royal Manchester Children's Hospital, Manchester, UK; 5 The Royal Hospital for Children, Glasgow, UK; 6 The Royal Hospital for Sick Children, Edinburgh, UK; 7 Great North Children's Hospital, Newcastle Hospitals NHS Foundation Trust, Newcastle upon Tyne, UK; 8 Institute of Cellular Medicine, Newcastle University, Newcastle upon Tyne, UK; 9 Arthritis Research UK Centre for Adolescent Rheumatology, GOS Institute of Child Health, University College London, London, UK; 10 Paediatric Rheumatology, Great Ormond Street Hospital NHS Foundation Trust, London, UK; 11 Arthritis Research UK Centre for Genetics and Genomics, The University of Manchester, Manchester, UK

**Keywords:** Juvenile Idiopathic Arthritis, Disease Activity, Outcomes research

## Abstract

**Objectives:**

Many criteria for clinically inactive disease (CID) and minimal disease activity (MDA) have been proposed for juvenile idiopathic arthritis (JIA). It is not known to what degree each of these criteria overlap within a single patient cohort. This study aimed to compare the frequency of MDA and CID across different criteria in a cohort of children with JIA at 1 year following presentation.

**Methods:**

The Childhood Arthritis Prospective Study recruits children at initial presentation to paediatric or adolescent rheumatology in seven UK centres. Children recruited between October 2001 and December 2013 were included. The proportions of children with CID and MDA at 1 year were calculated using four investigator-defined and eight published composite criteria. Missing data were accounted for using multiple imputation under different assumptions.

**Results:**

In a cohort of 1415 children and adolescents, 67% patients had no active joints at 1 year. Between 48% and 61% achieved MDA and between 25% and 38% achieved CID using published criteria. Overlap between criteria varied. Of 922 patients in MDA by either the original composite criteria, Juvenile Arthritis Disease Activity Score (JADAS) or clinical JADAS cut-offs, 68% were classified as in MDA by all 3 criteria. Similarly, 44% of 633 children with CID defined by either Wallace's preliminary criteria or the JADAS cut-off were in CID according to both criteria.

**Conclusions:**

In a large JIA prospective inception cohort, a majority of patients have evidence of persistent disease activity after 1 year. Published criteria to capture MDA and CID do not always identify the same groups of patients. This has significant implications when defining and applying treat-to-target strategies.

## Introduction

Juvenile idiopathic arthritis (JIA) represents the most common inflammatory rheumatic disease of childhood.^1^ To minimise pain and disability associated with active disease, one goal for all children with JIA is clinically inactive disease (CID), meaning absence of active inflammation.^2^ However, CID is not always achievable and a more realistic target may be minimal disease activity (MDA), meaning limited evidence of active inflammation.^3^ Defining either of these states in such a heterogeneous disease is challenging; there is no single diagnostic test for either state and as such multiple criteria have been proposed.

Simple clinical criteria for CID include no active joints or a score of zero on the physician (PGA) or parental (PGE) global evaluation. These single targets are easy to apply in clinical practice as part of the core outcome criteria for JIA.^4^ However, each alone may not capture the full spectrum of disease. Composite disease activity scores are more precise than their individual components, capturing multiple domains of disease activity and potentially increasing the statistical power of clinical trials.^5^ Over the past 15 years, a number of composite criteria have been proposed and variably validated in JIA patient populations. These include Wallace's preliminary criteria for CID and remission on and off medication,^2^ the Juvenile Arthritis Disease Activity Score (JADAS) and clinical JADAS (cJADAS) cut-offs for MDA, CID and remission^6^ ^7^ and the American College of Rheumatology (ACR) preliminary criteria for CID^8^ (table 1).

**Table 1:** Published CID and minimal disease activity criteria for JIA

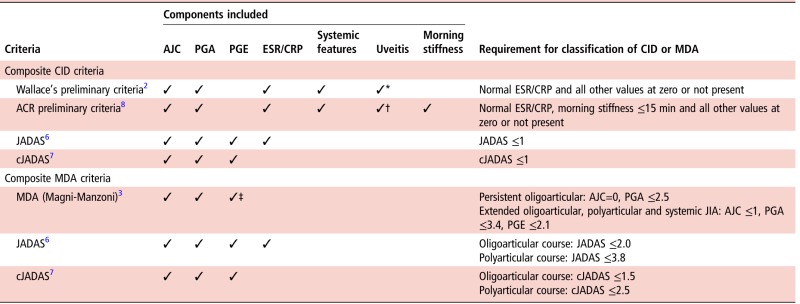

*Inactive uveitis was not defined.

†Inactive uveitis as defined by the Standardization of Uveitis Nomenclature working group.^15^

‡Not required for persistent oligoarticular JIA.

ACR, American College of Rheumatology; AJC, active joint count; CID, clinically inactive disease; cJADAS, clinical JADAS; CRP, C reactive protein; ESR, erythrocyte sedimentation rate; JADAS, Juvenile Arthritis Disease Activity Score; JIA, juvenile idiopathic arthritis; MDA, minimal disease activity; PGA, Physician's global assessment of disease activity; PGE, parental/child global evaluation of disease activity.

The aim of developing criteria for CID and MDA has been to better define the states of low disease activity as well as standardise outcome criteria across clinical trials and observational research.^2^ However, differences in patients identified by each set of criteria may contribute to the large variation in CID achievement described between cohorts observed in the literature.^9^ One study compared the frequency of CID according to a modified ACR preliminary criteria^8^ against achieving no active joints or zero on the PGA, PGE or child global assessments of disease activity. Within this single population, achievement of CID according to each set of criteria ranged from 19% to 68%.^10^ None of these single or modified composite criteria used in the study described has been validated in JIA and to date, no studies have directly compared published criteria within a single population to understand if they define similar groups of children.

Multiple high-quality studies support the efficacy of accelerated or targeted early treatment pathways in adult inflammatory arthritis (particularly rheumatoid arthritis (RA)).^11–14^ Treat-to-target approaches should lead to similar improvements in clinical outcomes for patients with JIA, but require valid, feasible and consistent treatment targets across different studies.

This study aims to apply single and published composite criteria for CID and MDA in a single patient population at a common time point: 1 year following initial presentation to rheumatology. The proportions of children reaching these states could then be compared between criteria of CID and MDA. Specifically, the study objectives are to (1) estimate the frequency of CID and MDA at 1 year following initial presentation; (2) investigate the differences in achievement of these disease states across International League of Associations for Rheumatology (ILAR) subtypes and (3) investigate if similar groups of children are captured by the different CID/MDA criteria.

## Methods

### Study population

Children and young people were participants in the Childhood Arthritis Prospective Study (CAPS), a UK multicentre inception cohort established in 2001. Details of this cohort have been described previously.^16^ To date, the cohort exceeds 1500 patients with childhood-onset inflammatory arthritis. CAPS was approved by the Northwest Multicentre Research Ethics Committee and written consent from parents/guardians was attained for all participants. Where possible, children also provided assent.

Patients were included in the current study if they had a physician diagnosis of JIA and had been recruited to the cohort before December 2013, to allow at least 1 year of follow-up. Prevalent cases and children with no returned study report forms were excluded.

### Data collection

The baseline date was that of first presentation to paediatric or adolescent rheumatology in one of seven centres in the UK. Baseline and 1-year follow-up data were collected from medical case notes, nurse and parent/patient questionnaires. Age and gender were recorded at baseline. Data from medical case notes included rheumatological diagnosis and ILAR subtype. ILAR category was defined using study data available at 1 year to allow children to ‘settle’ into a category. If ILAR category was missing at 1 year, the most recent previously collected ILAR category was used. Data from case notes also included number of active and limited joints (maximum 71), a 10 cm visual analogue scale (VAS) assessing physician's global assessment of disease activity, extra-articular disease features, results of laboratory investigations including erythrocyte sedimentation rate (ESR) (mm/hour) and C reactive protein (mg/L) and medication details including both antirheumatic and other therapeutics. Parent questionnaires included a Childhood Health Assessment Questionnaire, and two 10 cm VAS measures for pain and well-being, respectively. Young people over the age of 11 have the option to self-report.

### Outcomes of CID and MDA

Outcomes comprising a single criterion included no active joints and zero on the PGA or PGE. Published composite outcomes for the current analysis included CID according to the Wallace's preliminary criteria,^2^ JADAS^6^ and cJADAS^7^ and MDA according to the JADAS,^6^ cJADAS^7^ and the original Magni-Manzoni criteria^3^ (table 1). The sets of criteria were applied in full across all ILAR subtypes. As CAPS did not capture daily morning stiffness over most of the period of recruitment to this study, the ACR preliminary criteria for CID^8^ were not applied.

### Statistical analyses

All outcomes were calculated from extracted data items at the 1-year follow-up visit. The frequency and proportion of outcomes in all patients and within ILAR subtypes were reported. In addition, the overlap between patient groups identified by multiple outcomes was explored.

The primary analysis assumed that some data were missing (table 3) in a ‘missing-not-at-random’ mechanism. Children with missing data were split into six groups: those (1) discharged ‘well’, (2) discharged following repeat non-attendance, (3) transferred to other clinics, (4) moved home address or unknown reason for discharge, (5) lost to follow-up in CAPS and (6) follow-up form completed but with incomplete data. The following assumptions were made regarding these groups: patients were in CID according to all outcome criteria (groups one, two, five and three if transferred to adult services) or patients had normal laboratory criteria with other missing data missing at random (MAR) (group six). Unless assumed ‘well’, all other missing data were imputed via multiple imputation over 20 iterations assuming data MAR.

Secondary analyses included a complete case analysis as well as a most extreme scenario analysis, which assumed all children with missing data or forms were either entirely in active disease or entirely in CID/MDA for each set of criteria.

## Results

### Patient cohort

Up to December 2013, 1510 patients had been recruited to CAPS. Of these, 95 were excluded (60 were not diagnosed with JIA, 3 were prevalent cases and 32 had no available data), leaving 1415 children for analysis.

Sixty-five per cent of children were female and median age at first presentation was 8 years (IQR 3.5–12 years). The most common ILAR subtypes were oligoarticular (50%) and rheumatoid factor (RF)-negative polyarticular JIA (21%) (table 2). Median baseline active joint count was two (IQR 1–6) with median physician global assessment at 2.9 cm (IQR 1.5–5.0) (table 2).

**Table 2:** Baseline and 1 year characteristics of the cohort

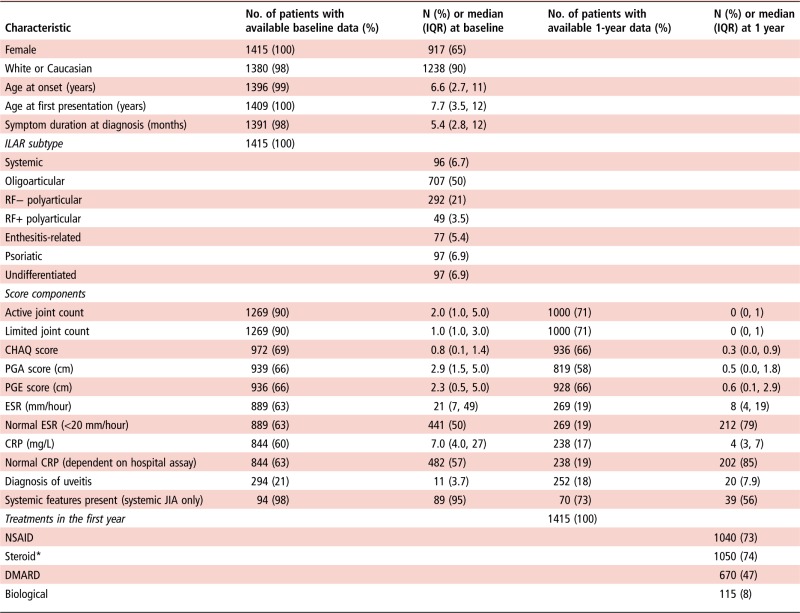

Data are presented as n (%) or median (IQR) where appropriate.

*Steroids administered orally/intravenous/intra-articular.

CHAQ, Childhood Health Assessment Questionnaire; CRP, C reactive protein; DMARD, disease-modifying antirheumatic drug; ESR, erythrocyte sedimentation rate; ILAR, International League of Associations for Rheumatology; JIA, juvenile idiopathic arthritis; NSAID, non-steroidal anti-inflammatory drug; PGA, physician global assessment of disease activity; PGE, parental global evaluation of disease activity; RF, rheumatoid factor.

### Achievement of CID or MDA

The 1-year follow-up form was not completed in 85 children. Fifty-nine had been discharged from rheumatology within the first year, including 24 who had been discharged ‘well’. Others had moved to another paediatric or adolescent clinic (n=11), moved to adult services (n=11) failed to attend (n=30) or were lost to follow-up (n=9). These patients did not differ significantly at baseline from those with 1-year data available, except for PGA score (available median PGA 2.9 cm, IQR 1.6–5.1, missing median PGA 2.0 cm, IQR 1.1–3.1, p=0.004).

Overall, 76% (95% CI 73 to 79) of patients achieved CID or MDA according to at least one set of criteria, with estimates ranging from 25% using Wallace's preliminary criteria to 67% if only an active joint count was used. Using composite criteria, fewer children achieved CID (range 30%–38%) compared with MDA (range 48%–61%) (table 3). Imputed estimates consistently exceeded those from complete case analysis (table 3).

**Table 3:** The frequency of CID and MDA using complete case and multiple imputation analyses

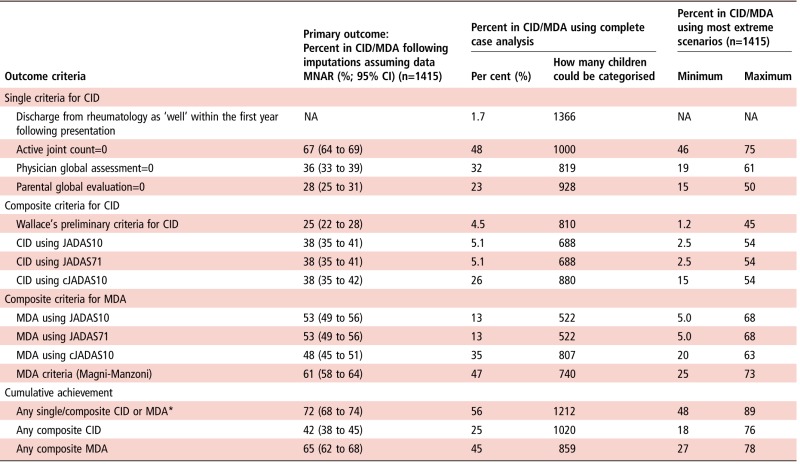

*Not including discharged ‘well’.

CID, clinically inactive disease; cJADAS, JADAS excluding erythrocyte sedimentation rate; JADAS, Juvenile Arthritis Disease Activity Score; MDA, minimal disease activity; MNAR, missing-not-at-random; NA, not available.

#### Estimates of CID across criteria

**Figure 1:**
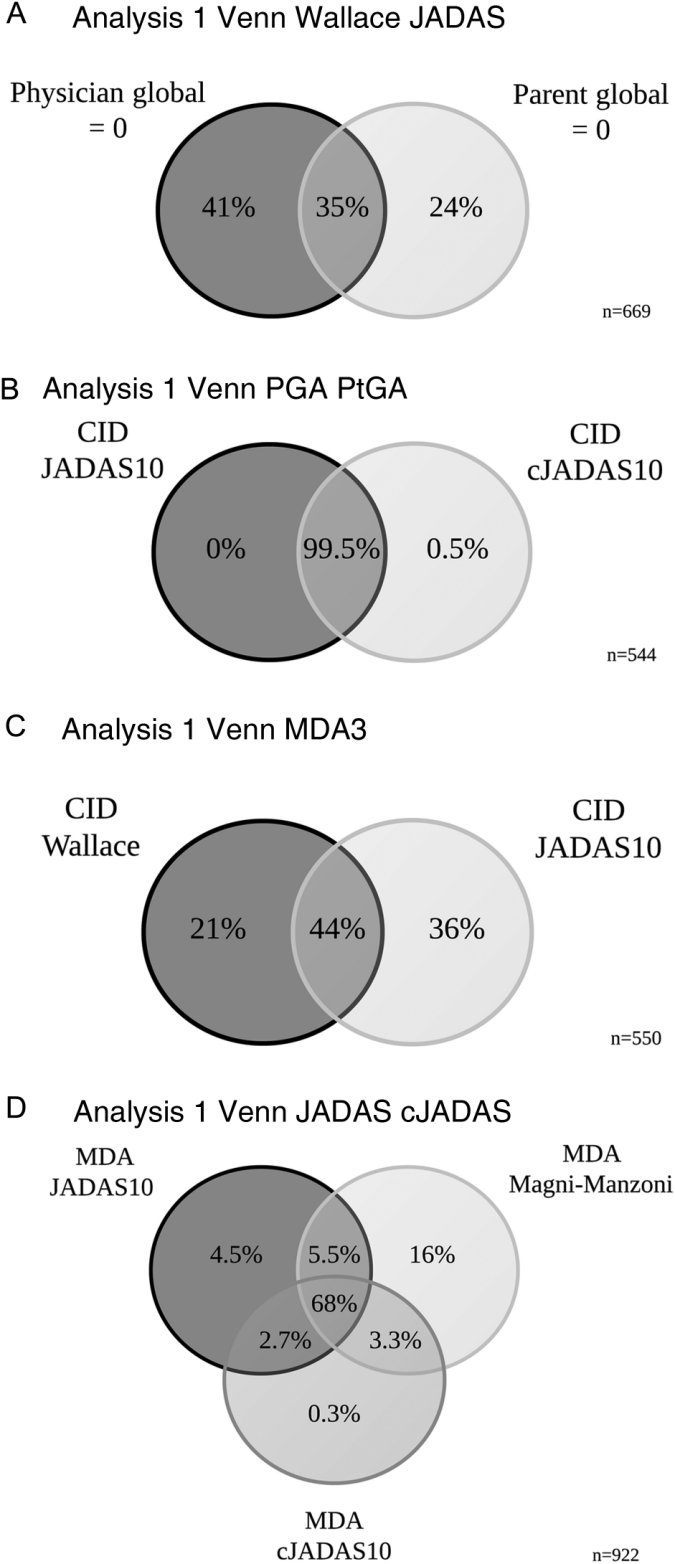
Per cent patient overlap between outcome criteria. (A) Zero on the PGA versus PGE, (B) CID JADAS10 versus cJADAS10, (C) CID Wallace's preliminary criteria versus JADAS10 and (D) MDA Magni-Manzoni, JADAS10 and cJADAS10. For each figure, percentages are out of all children who satisfied at least one of the criteria displayed. ESR, erythrocyte sedimentation rate; cJADAS: clinical JADAS excluding ESR; CID, clinically inactive disease; JADAS10, Juvenile Arthritis Disease Activity Score in 10 joints; MDA, minimal disease activity.

At 1 year following initial presentation to rheumatology, 42% (95% CI 38% to 45%) of patients satisfied at least one of the composite criteria for CID. This is in contrast with 67% (95% CI 64% to 69%) that had achieved an active joint count of zero. A third of children had no active joints, but did not achieve a score of zero on the PGA (33%). Rarely a child with active joints was scored at zero on the PGA (4%). In these few cases, children had only one active joint and appeared well on other disease variables (table 4). Physicians and parents appeared to score differently with only 35% overlap in patients scoring zero on both the PGA and PGE (figure 1A). The JADAS and cJADAS criteria, which include a mixture of physician and parent-assessed measures, had a high degree of overlap (almost 100%) and both identified 38% of children as in CID at 1 year (table 3 and figure 1B). Discrepancies between these two groups were driven by ESR, with patients (n=3) in CID only on the cJADAS with median 42 mm/hour ESR (IQR 34–65 mm/hour), compared with 6 mm/hour (IQR 2–11 mm/hour) for those in CID on both criteria (table 4).

**Table 4:** Comparison of disease activity where CID/MDA criteria were discordant

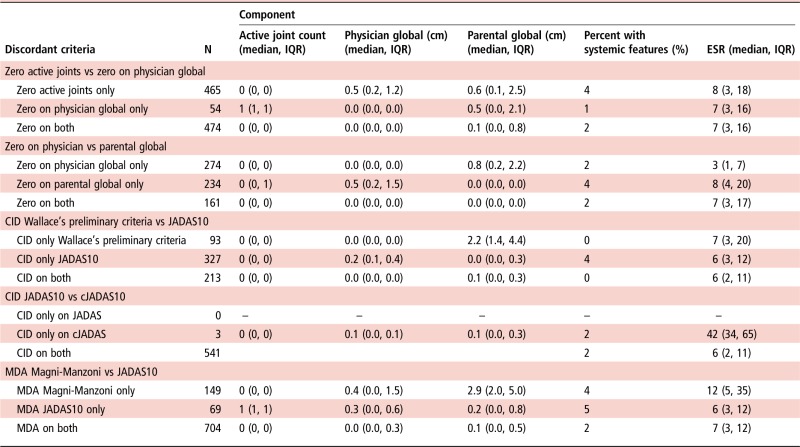

CID, clinically inactive disease; cJADAS, clinical JADAS; ESR, erythrocyte sedimentation rate; JADAS10, Juvenile Arthritis Disease Activity Score weighed to 10 joints; MDA, minimal disease activity.

Fewer patients achieved CID on Wallace's preliminary criteria (25%, contains only physician-assessed components) compared with the JADAS criteria (38%, contains both physician and parent-assessed components). In accordance, there was only a 44% overlap in the patients identified in CID by these criteria (figure 1C). Where discordances existed between patients defined as in CID on the Wallace's preliminary criteria only or the JADAS10 only, large differences in the PGE (median 2.7 and 0.0 cm, respectively) were evident. However, patients in CID on the cJADAS had median PGA of 0.2 cm, highlighting the requirement of an absolute cut-off of 0 cm required for the Wallace's preliminary criteria. In addition, 4% of patients with systemic JIA in CID on the JADAS10 were recorded as having had active systemic features (table 4). There were no differences in active joint count or ESR between these groups.

#### Estimates of MDA across criteria

At 1 year following presentation, 65% (95% CI 62% to 68%) of patients satisfied at least one of the three MDA criteria. The range in proportion of children achieving this state was smaller than using CID criteria, ranging from 48% on the cJADAS to 61% using the Magni-Manzoni criteria (table 3). There was also greater overlap between MDA criteria than CID with 68% of children classified in MDA by all three criteria. The largest discrepancy was for the Magni-Manzoni criteria, which does not require use of the PGE score in oligoarticular JIA (figure 1D). In accordance, median PGE scores were higher in patients in MDA only on the Magni-Manzoni criteria compared with the JADAS10 (2.9 cm, IQR 2.0–5.0 cm vs 0.2 cm, IQR 0.0–0.8) (table 4).

### The frequency of CID and MDA in each ILAR subtype

**Figure 2:**
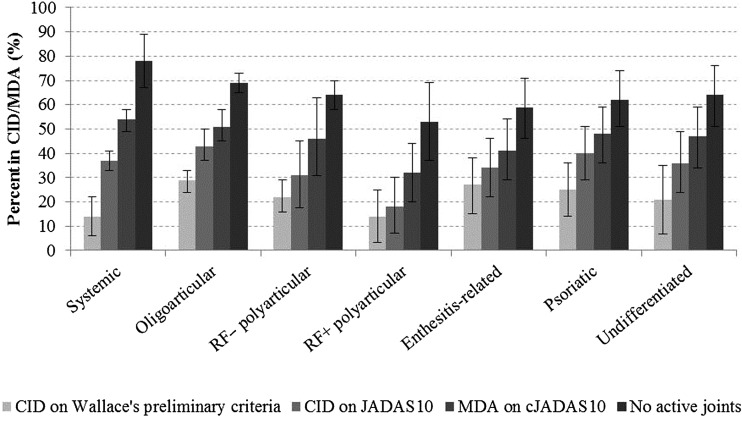
Per cent of patients with juvenile idiopathic arthritis (JIA) who had achieved CID and MDA states at 1 year following presentation. CID, clinically inactive disease; MDA, minimal disease activity; JADAS, Juvenile Arthritis Disease Activity Score; cJADAS, clinical JADAS; RF, rheumatoid factor.

A similar pattern across outcome criteria was seen across all ILAR categories, with patients achieving no active joints more frequently than MDA, CID on JADAS and CID on Wallace's preliminary criteria, respectively (figure 2). However, achievement of the most stringent composite criteria was achieved most frequently in the oligoarticular subtype (CID Wallace's preliminary criteria: 29%, CID JADAS10 43%). Patients with systemic JIA experienced large variation in their outcomes, achieving fewer of the criteria sets when more information on systemic features was taken into account. These children had high achievement of no active joints (78%), but only 14% achieved CID on Wallace's preliminary criteria (see figure 2 and online supplementary table S1).

10.1136/annrheumdis-2016-210511.supp1 supplementary data

## Discussion

Since the success of treat-to-target approaches in RA,^17^ ^18^ similar strategies in JIA have been considered.^19–22^ However, many published and investigator-defined targets have been used across research.^9^ ^23^ ^24^ The current study highlights that even published targets intended to capture the same construct identify different groups of children. Results from studies using different outcome criteria therefore cannot be compared directly. In addition, if used as targets in clinical practice, using different targets may result in overtreatment or undertreatment.

Achievement of CID and MDA varied greatly between criteria in this cohort. Broad achievement of CID was around 30% and MDA around 50%. This indicates a significant level of ongoing disease symptoms at 1 year following initial presentation. Using Wallace's preliminary criteria, only 25% children achieved CID. Higher estimates have been gained from other inception cohorts within similar timeframes in the literature, with estimates of 45% within 1 year^25^ and 53% at 18 months.^26^ These higher estimates are likely artefacts of measuring ever achievement in the former study and not including children with polyarticular JIA in the latter. Estimates of CID from the literature not using Wallace's preliminary criteria vary widely from 19%^27^ to 60%.^28^

Estimates of CID using newer criteria, including cut-offs on the JADAS^6^ or cJADAS^7^ have not yet been published from other inception cohorts. The current study found that the JADAS and cJADAS CID cut-offs have high overlap, capturing almost identical groups of children. Where discordant, children only in CID on the cJADAS had substantially higher ESR (42 vs 0 mm/hour). That so few children presented with low symptomatology but high inflammatory markers suggests a non-rheumatological cause of high ESR, such as recent infection. The cJADAS was designed to be more feasible in clinical practice compared with JADAS, since ESR is not required.^29^ Since overlap between these criteria was excellent and complete data were available in 20% more patients in the three variable cJADAS, the current data support the use of cJADAS in preference to JADAS when assessing CID. However, overlap between the JADAS and Wallace's preliminary criteria was poorer.

Lower overlap between the physician and parent global assessments, and between Wallace's preliminary criteria and the JADAS criteria, may reflect the different components included within each criteria and challenges the concept of what constitutes inactive or minimal disease in JIA. Unlike Wallace's preliminary criteria, the JADAS and cJADAS include a subjective parent (child)-assessed component (table 1). Overlap between this and either JADAS set of criteria was only 44% (figure 1). The minimally different median scores on the PGA for children in CID only on Wallace's preliminary criteria (0.0 cm) versus only the JADAS10 (0.2 cm) suggest that clinicians may not mark children at exactly zero, even on resolution of active disease. In this study, scores were recorded on paper and transcribed into the study database. With a move to online data capture and electronic medical records, this issue may resolve if relating to transcription errors, but equally it may be that clinicians did not feel they could mark at exactly zero. These issues are resolved when using criteria such as the JADAS or cJADAS. Since CID on these numeric rating scales is defined as any score lower or equal to one, minimal scores above zero will be captured as part of the spectrum of CID. However, the substantial difference in the PGE (medians 2.2 and 0.0 cm, respectively) suggests a marked difference in global well-being for children identified as CID on the different criteria. The patient global assessment has been shown to be driven in large part by ongoing pain.^30^ ^31^ A feature of JIA for a subset of children is the resolution of inflammation with persistent pain symptoms, which patients themselves have considered as active disease.^32^ While it is not possible to disentangle pain, related to inflammation or not, from other active disease symptoms using patient and parent global assessments, any symptom that patients themselves feel relate to their disease and require treatment via rheumatology should be treated as such. In concordance, applying criteria such as the JADAS and cJADAS which assess both inflammation and a patient's assessment of their disease may identify children with persistent chronic pain independent of joint inflammation, particularly in cases where scores are high despite the absence of active joints. These children could then be targeted for alternative pain management strategies, for example, psychological support.

ILAR subtype-specific estimates provided some evidence that patients with the less common subtypes are less likely to achieve CID. This may be a result of higher PGA ratings for patients with extra-articular features, including exanthema and macrophage activation syndrome for systemic JIA. For the majority of criteria, oligoarticular JIA was the most favourable disease course with RF-positive polyarticular the least favourable, corroborating existing evidence.^25^ ^33–35^ Patients with systemic disease had the largest variation in outcome estimates (14%–78%), which was largely driven by components of the individual composite criteria; when more information on systemic features was included, fewer children in this subtype achieved the outcome. This trend indicates that systemic features should be included when assessing CID or MDA in children with systemic JIA and highlights the importance of both physician-reported and patient/parent-reported global scores. Since no sets of criteria included in this analysis explicitly captured enthesitis or psoriasis, it remains to be seen whether the inclusion of criteria based on these features improves the measurement of CID in affected children.

The current study benefits from studying a large inception cohort of patients across all ILAR subtypes of JIA. It highlights the difference in disease states being targeted by clinicians and used as outcomes in research. Going forward, the introduction of composite measures and treatment targets into the clinical setting have the potential to streamline the collection of clinical data, enabling comparisons from one visit to the next and between different centres. Further work is indicated to establish the feasibility, acceptability and utility of composite scores and treatment targets in clinical practice as well as in the context of clinical trials to improve future data capture and completeness. This study also provides an update on the frequency of CID and remission in a contemporary JIA cohort. This information is very important in the clinical setting, helping clinicians to realistically manage patient expectations at presentation. As the primary outcome was overlap between the published definitions, which is unlikely to change over time, multiple time points were not assessed. A previous study in this cohort, however, assessed achievement of CID on the cJADAS71 across children with JIA who initially presented to paediatric rheumatology between 2001 and 2011. They reported no significant increase in CID achievement for children presenting in later years, despite a wider variety of biological availability and a culture of more aggressive treatment strategies.^21^ ^36^

Limitations of the current study include that CID criteria were applied to ILAR categories in which they are not validated: namely systemic, enthesitis-related and psoriatic JIA. However, the majority of previous studies have applied CID criteria in their entire cohort including all categories.^37–41^ Therefore, to assess the same disease state as applied in existing literature and allow comparisons across all outcomes, the criteria were applied to all JIA subtypes. As a consequence, current estimates may overestimate the frequency of CID in children with persistent systemic manifestations, enthesitis or psoriasis but no active joint inflammation. Since these features are relatively rare, estimates across the entire cohort were likely only marginally affected by their inclusion and these features are at least partially reflected in physician and parental global scores. ILAR subtype-specific definitions of CID may be of value in the future.

In this study, all items were captured as part of routine care and not specifically within the setting of a clinical trial or study, and many of the criteria included were not designed for a busy clinical setting.^42^ The capture of these items in routine clinical care of JIA vary greatly between clinical settings and composite measures of disease activity were not routinely collected in UK practice during the time of data capture in this study. This is reflected in part by the amounts of missing data, a common observation in ‘real-world’ research studies. The volume of missing data was particularly high for composite measures, where multiple elements were often not collected routinely. We account for these missing data using a number of assumptions and multiple imputation. Estimates using complete case analyses were substantially lower than after imputation, partly due to children with active disease being easier to classify in the complete case analysis (see online supplementary material). The majority of data missing were for laboratory measures, with only 20% of children having ESR recorded at 1 year, reflecting that children who are well may not have blood tests. Fantini *et al* also reported that patients lost to follow-up had greater remission rates than those present at follow-up.^33^ These trends likely biased complete case estimates towards a greater proportion of children with ongoing disease activity.

The frequency of remission according to the 2011 ACR preliminary criteria^8^ could not be calculated in the current study. This set of criteria requires information on morning stiffness, which is notoriously difficult to determine in young children and which was not collected as part of CAPS. International consensus should be reached about a minimal core data set for both observational and interventional research. This would aid monitoring of the JIA disease course and response to therapies, and also aid comparability between clinical research studies. Where feasible, future work should compare the frequency of achieving the CID on the ACR 2011 preliminary criteria, with the states highlighted in the current study.

Finally, this study highlights that published definitions of CID and MDA identify distinctly different groups of children. While the cJADAS10 cut-offs are more feasible to apply in clinical practice and appear to capture a greater picture of active JIA compared with Wallace's preliminary criteria, this study does not provide data to support which measure is optimal in terms of long-term outcomes. Future studies should compare long-term outcomes following early achievement of these measures to provide evidence for a potential aim for treat-to-target approaches. Currently, there are no recommendations for optimal treat-to-target strategies and as such, these strategies are not common practice.

## Conclusion

In a large inception cohort, a large proportion of patients with JIA had evidence of persistent disease activity 1 year following first presentation to paediatric or adolescent rheumatology. However, the estimates of these disease states differed widely based on which set of criteria was applied, many of which were not disease subtype-specific. These differences highlight that the same child could be classified as ‘in CID’ or having active disease at the same time point between clinicians or hospitals. Future work needs to explore which treatment target predicts better long-term prognoses in JIA.
