# Supplementary material for: How common is clinically inactive disease in a prospective cohort of patients with juvenile idiopathic arthritis? The importance of definition
Source: Ann Rheum Dis. 2017 Apr 7;76(8):1381–8. doi: 10.1136/annrheumdis-2016-210511 (PMC5738598; doi:10.1136/annrheumdis-2016-210511)
Supplement: supplementary data [file annrheumdis-2016-210511supp001.pdf]

SUPPLEMENTARY MATERIALS

Methods for handling missing data

Outcome frequencies were assessed using complete case analysis in a secondary analysis. This included children for whom a complete dataset was not available but the child could still be classified as ‘not in CID/MDA’ if one available component excluded that possibility. For example, a child scoring 2cm on the PGA could not be in CID on any set of criteria. This child was classified as ‘not in CID’, even if other missing data existed.

All imputation models were completed under the same random number seed (5879). Variables entered into the imputation models included hospital, gender, age, disease duration at presentation, active joint count, PGA, PGE, ESR, CRP, CHAQ, ILAR subtype and non-steroid anti-inflammatory drug, steroids or disease-modifying anti-rheumatic drug prescription (yes/no). Continuous variables were transformed to normal distributions before imputation [41], after which they were converted back to their original forms. Composite criteria estimates were calculated using individual components following imputations.

Supplementary Table 1. The frequency of CID/MDA in each ILAR subtype using multiple imputation assuming data MNAR

ID criteria	Percent of patients in CID/MDA at year one of follow-up using Multiple Imputation assuming data MNAR (%) (95% CI)						
	**Systemic (n=96)**	**Oligo (n=707)**	**RF- Poly (n=292)**	**RF+ Poly (n=49)**	**ERA (n=77)**	**PsA (n=97)**	**Undiff. (n=97)**
**Single criteria for CID**							
Active joint count = 0	78 (67, 89)	69 (65, 73)	64 (58, 70)	53 (37, 69)	59 (46, 71)	62 (51, 74)	64, 51, 76)
Physician global assessment = 0	32 (21, 43)	40 (35, 45)	30 (24, 36)	18 (5.6, 31)	36 (24, 47)	39 (28, 51)	36 (21, 51)
Parental global evaluation= 0	29 (19, 40)	32 (28, 36)	19 (14, 25)	14 (2.7, 26)	25 (14, 36)	25 (14, 37)	30 (19, 42)
**Composite criteria for CID**							
Wallace's preliminary criteria for CID	14 (6.1, 22)	29 (24, 33)	22 (16, 29)	14 (3.3, 25)	27 (15, 38)	25 (14, 36)	21 (6.7, 35)
CID using JADAS10	37 (26, 49)	43, 39, 47)	31 (25, 38)	18 (4.6, 32)	34 (22, 46)	40 (29, 51)	36 (24, 49)
CID using JADAS71	37 (26, 49)	43 (39, 47)	31 (25, 38)	18 (4.6, 32)	34 (22, 46)	40 (29, 51)	36 (24, 49)
CID using cJADAS10	38 (26, 49)	43 (39, 48)	32 (26, 38)	18 (4.6, 32)	34 (22, 46)	40 (29, 51)	36 (24, 49)
**Composite criteria for MDA**							
MDA using JADAS10	53 (42, 64)	52 (48, 57)	57 (50, 63)	44 (28, 61)	46 (32, 60)	51 (40, 63)	53 (40, 66)
MDA using JADAS71	53 (42, 64)	52 (48, 57)	57 (50, 63)	44 (28, 61)	46 (32, 60)	51 (40, 63)	53 (40, 66)
MDA using cJADAS10	54 (42, 66)	51 (46, 55)	46 (40, 53)	32 (17, 49)	41 (29, 54)	48 (36, 59)	47 (34, 59)
MDA (Magni-Manzoni)	59 (49, 70)	69 (65, 73)	52 (46, 59)	38 (22, 53)	53 (40, 66)	59 (47, 71)	59 (46, 71)

MNAR: missing not at random; Oligo: oligoarticular JIA; RF- Poly: RF negative polyarticular JIA, RF+ poly: RF positive polyarticular JIA, ERA: Enthesitis-related JIA, PsA: Psoriatic JIA, Undiff. Undifferentiated JIA. Pers: Persistent; Ext: Extended; CID: Clinically inactive disease; MDA: Minimal disease activity; JADAS: Juvenile arthritis disease activity score in 10 (JADAS10) and 71 (JADAS71) joints and excluding ESR (cJADAS10).

Supplementary Table 2. The frequency of CID/MDA in each ILAR subtype using Complete Case analysis

Outcome	Percent of patients in CID/MDA at year one of follow-up using Complete Case analysis (%)							
	**No. missing (%)**	**Systemic (max n=90)**	**Oligo (max* n=663)**	**RF- Poly (max n=282)**	**RF+ Poly (max n=47)**	**ERA (max n=72)**	**PsA (max n=94)**	**Undiff. (max n=76)**
**Single criteria for CID**								
Discharge from rheumatology due to low disease activity	66 (4.7)	1 (1.1)	9 (1.4)	2 (0.7)	0 (0.0)	1 (1.4)	1 (1.1)	2 (2.6)
Active joint count = 0	415 (29)	50 (55)	253 (38)	102 (36)	11 (22)	22 (30)	32 (34)	10 (13)
Physician global assessment = 0	596 (42)	15 (28)	145 (35)	57 (30)	5 (15)	17 (33)	21 (36)	5 (25)
Parental global evaluation = 0	491 (35)	16 (26)	134 (29)	31 (15)	4 (12)	8 (16)	11 (17)	11 (24)
**Composite criteria for CID**								
Wallace's preliminary criteria for CID	514 (39)	2 (3.2)	11 (3.0)	16 (7.8)	4 (9.8)	2 (4.2)	1 (1.6)	0 (0.0)
CID using JADAS10	638 (48)	5 (9.8)	13 (4.3)	10 (5.8)	0 (0.0)	1 (2.4)	5 (9.4)	1 (3.2)
CID using JADAS71	638 (48)	5 (9.8)	13 (4.3)	10 (5.8)	0 (0.0)	1 (2.4)	5 (9.4)	1 (3.2)
ID using cJADAS10	708 (53)	16 (39)	130 (42)	46 (32)	3 (13)	12 (30)	19 (45)	6 (40)
**Composite criteria for MDA**								
MDA using JADAS10	887	7 (18)	23 (9.7)	25 (20)	3 (13)	3 (8.6)	7 (16)	2 (10)
MDA using JADAS71	887	7 (18)	23 (9.7)	25 (20)	3 (13)	3 (8.6)	7 (16)	2 (10)
MDA using cJADAS10	602	3 (13)	7 (18)	23 (9.7)	25 (20)	3 (8.6)	7 (16)	2 (10)
MDA criteria (Magni-Manzoni)	545	25 (46)	271 (62)	82 (42)	7 (22)	24 (44)	34 (52)	12 (41)

*The sample sizes represent the total number of children with these subtypes. However, missing data may have been evident so that the total number will not have been categorised by each set of CID criteria. Oligo: oligoarthritis; RF- poly: Rheumatoid factor negative polyarthritis; RF+ poly: Rheumatoid factor positive polyarthritis; ERA: Enthesitis-related arthritis; PsA: Psoriatic arthritis; Undiff: Undifferentiated arthritis; CID: Clinically inactive disease; MDA: Minimal disease activity; JADAS: Juvenile arthritis disease activity score weighted to 10 (JADAS10) and 71 (JADAS71) joints and excluding ESR (cJADAS10).
